# Nurses’ perceptions, attitudes, and perspectives in relation to climate change and sustainable healthcare practices: A systematic review

**DOI:** 10.1016/j.joclim.2023.100290

**Published:** 2023-12-02

**Authors:** Ebenezer Akore Yeboah, Amanda Rodrigues Amorim Adegboye, Rosie Kneafsey

**Affiliations:** a Research Centre for Healthcare and Communities, Coventry University, UK; b Centre for Agroecology, Water and Resilience, Coventry University, UK

**Keywords:** Nursing, Climate change, Environmental sustainability, Environmental responsible healthcare, Net-zero healthcare, Sustainable healthcare

## Abstract

**Background:**

Climate change threatens human existence and is caused by increasing carbon emissions. Healthcare systems generate about 5% of global net CO2 emissions, further contributing to the crisis. Green healthcare practices could be implemented and nurses, as the largest workforce group, could potentially drive these practices. This review explored nurses’ awareness, perceptions, attitudes and perspectives towards sustainable nursing and healthcare practices concerning climate change.

**Methods:**

The Joanna Briggs Institute [JBI] methodology for conducting mixed methods systematic reviews was applied and results were reported following Preferred Reporting Items for Systematic Reviews and Meta-Analyses [PRISMA] guidelines. CINAHL, PsycINFO, SCOPUS, and PUBMED databases were searched. JBI and Mixed Method Appraisal Tool [MMAT] critical appraisal tools were used for the data appraisal. Data synthesis and integration followed the JBI convergent integrated approach and thematic analysis was performed. https://doi.org/10.17605/OSF.IO/8H3TC.

**Findings:**

Eighteen papers were included that represented nine different countries across five continents. One study was found in Africa, no studies in South America, and three in Asia. Five key themes were identified: i) knowledge and awareness of climate change, ii) link between nursing and climate change, iii) environmental sustainability, iv) barriers to environmentally responsible healthcare, and v) routes to environmentally sustainable nursing practices.

**Interpretation:**

The review indicates the need to raise awareness regarding climate change and sustainable practices among nurses. It is vital policy makers, and healthcare leaders ensure criteria relating to environmental sustainability and carbon reduction are included in decisions about procurement and service delivery. Nurses’ engagement could drive forward a net-zero agenda.

## Introduction

Known to be the single most serious threat to human survival, climate change is having a dramatic impact on humanity and the planet [1]. It has been defined by the United Nations [UN] as long-term shifts in temperature and weather patterns [2]. Variations in the solar cycle, volcanoes, orbital and geochemical cycle changes were identified as the major natural causes of climate change by scientists in the 19th century [3]. However, it is established through mathematical modelling and scientific techniques such as satellite retrieval that anthropogenic [human] activities are now the major causes of climate change. These activities include; the use of fossil fuels for energy production, coal for heating buildings, deforestation, and land use, to mention a few [3]. Climate change is creating changes in the survival conditions of humankind and having a negative impact on a range of health outcomes [4]. For example, global increases in food poverty and respiratory diseases [5] necessitate urgent attention [4] in addition to the growing populations of climate refugees forcibly displaced by famine, drought, flooding, and fires.

In response to climate change, the NHS-UK has committed to reaching net zero by 2040 via the emissions they control directly, and by 2045 those emissions they can influence [6]. The NHS is undertaking actions such as; electrification of the NHS transport fleet, construction of net zero hospitals, LED light replacement and, strategic supply change [6].

Healthcare professionals, particularly nurses have the potential to mitigate and advocate for climate actions collectively or as individuals [7]. Nurses are known to be consumers of a variety of materials in their daily activities, such as care products, pharmaceutical and nutritional products [8], [9], [10]. In many care settings, products may not be used, or partially used, expired and are wasted whilst a huge proportion of healthcare devices and equipment are designed to be single use only. Some consumables are overly packaged leading to an increased healthcare carbon footprint.

A number of environmentally responsible nursing practices have been reported in the literature such as shutting down computers when not in use, placing used items in appropriate bins, shared transport services, tele-conferencing to replace staff meetings [11], editing files electronically rather than printing, and using waste paper to print a draft [12]. Reduction of waste during care and asking leaders to help create a less polluted environment are also seen as sustainable practices [13]. In a study from China, not using disposable supplies, garbage classification, waste recycling, and green travel, which have positive effects on the planet were reported as sustainable practices among nurses [14].

However, research suggests that nurses may not be aware of the potential to effect positive action towards climate change [15]. For example, Swedish nurses had some understanding of local environmental activities, but limited knowledge of the global impacts of healthcare on climate change [16]. Similarly, Xiao et al., [2016] found that whilst nurses were aware of the impact of climate change on public health, there was little acknowledgement of the relationship between healthcare activities and climate impact [17]. These depict some gaps within the scope of nursing practice in relation to climate change.

To explore this topic further, a critical and systematic evaluation of the literature is required. Therefore, this mixed methods review explores nurses’ perceptions, attitudes, awareness, and perspectives towards sustainable nursing practices in relation to climate change with a view to informing nursing curriculum development and professional practice.

## Method

This systematic review followed the Joanna Briggs Institute [JBI] methodology for conducting a mixed-methods systematic review [18]. The Preferred Reporting Items for Systematic Reviews and Meta-Analyses [PRISMA] [19] was used for reporting the study. The review has been registered in Open Science Framework [OSF], https://doi.org/10.17605/OSF.IO/8H3TC.

### Searching literature

A preliminary scoping search was undertaken to inform the final search strategy. A full search strategy was developed after reviewing various titles and abstracts of relevant articles and the index terms used to describe the articles in PUBMED and later tailored appropriately for use in other databases and piloted prior to final searches. The databases searched included; CINAHL, PsycINFO, SCOPUS, and PUBMED. The inclusion criteria were empirical papers focused on registered nurses regardless of work setting across the globe [see supplementary material]. Reference lists of included papers were also searched manually. Forward citation searching was also performed. All studies regardless of the date of publication were included and were limited to publications in English.

### Study screening and selection process

**Fig. 1:**
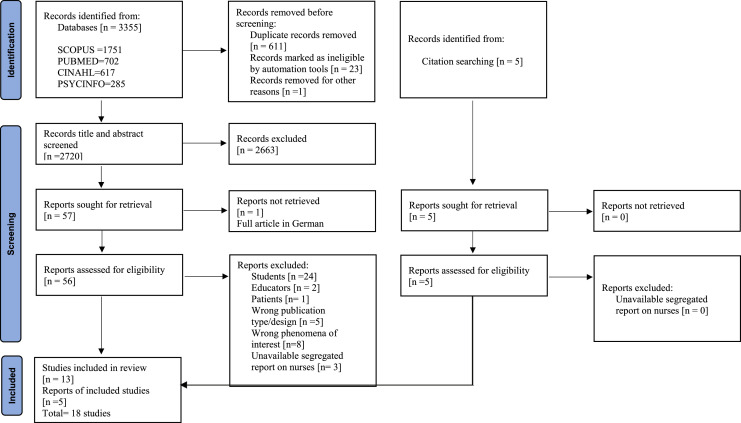
PRISMA 2020 flow diagram for nurses’ perceptions, attitudes, and perspectives in relation to climate change and sustainable healthcare practices.

The searched results from each database were imported into EndNote X9 [Bld 12,062] where duplicate citations were removed. The papers were subsequently transferred to Rayyan web tool [20] where further duplicate removal and screening processes by two reviewers [EAY, ARAA] took place based on the inclusion and exclusion criteria. Initial screening of approximately 30 % of the studies was done by three members [EAY, ARAA, RK] of the review team independently, based on titles and abstracts. Two members of the review team [EAY, ARAA] screened all the other studies, based on titles and abstracts. Four papers were found to be conflicting corresponding to less than 0.5 % disagreement and it was resolved through discussion. All studies included at this stage were then subjected to screening by full-text articles by the same two members of the review team and subsequently assessed for eligibility. Reasons for excluding a full text study that did not meet the inclusion criteria were recorded and presented in the PRISMA flowchart [Fig. 1] [21].

#### Assessment of methodological quality

The quantitative papers and mixed methods papers were appraised using the Mixed Methods Assessment Tool [MMAT] version 2018 [22]. The qualitative papers selected for retrieval were appraised using the JBI qualitative appraisal tool [23]. The quality appraisal was done by the first reviewer [EAY] and later assessed by the second reviewer [ARAA], with the third reviewer [RK] resolving issues of dissimilarity. All studies, regardless of their methodological quality, underwent data extraction and synthesis.

### Data extraction

Quantitative and qualitative data were extracted from the included studies by adapting the standardized JBI mixed methods data extraction form following a convergent integrated approach [18]. Information extracted from each included article was categorised into two; general characteristics [i.e., author, country, aim, sample and characteristics, and design] table 1, and findings with/without quotes depending on the study type, [see supplementary material]. Authors of four papers were contacted to request for independent findings on nurse data, and only one responded.

**Table 1:** General characteristics of included papers.

Author/Year	Country	Aim	Sample Characteristics	**Design**
Anaker, et al., [2015].	Sweden	Perceptions of climate and environmental issues and examine how nurses perceive their role.	18 nurses.	Qualitative, descriptive explorative study
Baid, et al., [2019].	England-UK	Explanation of the concept of sustainability in a critical practice from the practitioner's perspective.	11 healthcare professional [8 nurses, 2 PT, 1 technician].	Qualitative, Charmazian constructivist grounded theory approach
Kalogirou, et al. [2020].	Canada	Perspectives on climate change, health, nursing practice and the relationships between these concepts.	22 nurses	Focused ethnography
Kalogirou, et al., [2021].	Canada	How hospital context influences nurses’ abilities to promote and engage with environmentally responsible practice.	22 nurses	Focused ethnographic study
Iira, et al. [2021].	Finland	Perception of nurses’ preparation to address climate change health impacts	6 nurses,	Qualitative descriptive research method
Leppänen, et al., [2022].	Finland	How nurses and nurse managers consider sustainable development principles in their daily work, and decision-making.	26 nurses	Qualitative descriptive design with naturalistic orientation
Koltsida, et al. [2021].	Sweden	Experience of IT use in home health care through a sustainable development model	10 registered and District nurses	Qualitative design
Dunphy [2014].	Australia	Key obstacles to healthcare professionals supporting environmental sustainability	64 healthcare professionals and academics [nursing and midwifery=24 %]	Qualitative study
Xiao et al. [2016].	China	Knowledge and attitudes of nurses concerning climate change and nurses’ role in addressing its impacts.	330 registered nurses	Cross-sectional study
Polivka, et al., [2012].	USA	Knowledge and attitudes of public health nurses concerning climate change and their role.	Public health nursing administrators in all U.S. state and local health departments [*n* = 786]	Cross-sectional survey
Buriro, et al. [2018].	Pakistan	Knowledge, perception and information sources of nurses about climate change.	105 registered nurses, nursing managers and management officials.	Cross-sectional study
Li, et al. [2021].	China	Relationship between green behaviour intentions and green behaviour and the moderating role of ethical leadership.	489 nurses	Cross-sectional study
Schenk, et al., [2021].	USA	Awareness, experience, motivation, and behaviours related to climate change and health	489 participants [81 practicing nurses, students=255, faculty= 50]	Descriptive survey study
Barraclough, et al., [2019].	Australia	Environmental sustainability and guide future initiatives to reduce the environmental impact.	628 haemodialysis chairs [nurse unit managers].	Cross-sectional survey
May et al., [2019].	Pennsylvania, USA	Knowledge, attitudes, and behaviours of school nurses related to the health impacts of climate change	262 school nurses	Descriptive correlational study
Nieto-Cerezo, [2016].	UK	Attitudes to more sustainable mode of transport of patients, visitors and staff	230 travel surveys were completed by patients, visitors and staff	Case study
Nsengiyumva et al., [2020].	Rwanda	Awareness of climate change and perceptions of potential neonatal health risks associated with climate change	184 participants [nurses=107 and midwives].	Descriptive cross-sectional design
Kallio, et al., [2020].	Finland	Views on environmentally responsible clinical practices, and stakeholders’ roles and tools needed.	35 nursing staff	Modified Delphi method with two rounds. Mixed methods

### Data transformation, integration and analysis

In accordance to the JBI convergent integrated approach for mixed methods systematic reviews [18], the retrieved quantitative data underwent qualitizing thus transformation of reports into textual descriptions or narrative interpretation [24]. Qualitizing means extracting data from quantitative studies and translating or converting it into ‘textual descriptions’ to allow integration with qualitative data, in a way that answers the review question [45]. These textual descriptions are then assembled and pooled with the qualitative data extracted directly from qualitative studies [45].

During data extraction, the themes and quotes of relevance were taken from the qualitative papers for synthesis. The qualitized data were assembled with the qualitative data and then categorised into subthemes. The categorised data were pooled together based on similarity in meaning to produce a set of integrated findings [themes] [25]. The subthemes and themes were refined through discussion with review members [EAY,ARAA,RK] to establish an accurate reflection of the data. Thematic analysis was carried out and finally, a narrative account of the synthesis with quotes from included papers was presented.

## Results

The database search and citation searching retrieved 3360 articles. After the removal of duplicates, 2752 articles were subject to abstract and title screening, 62 papers were eligible for full-text screening and 18 papers met the inclusion criteria. The 18 included studies comprised nine quantitative, eight qualitative and one mixed methods paper. The articles represented studies from nine different countries across five continents, with the majority from Europe [*n* = 7], North America [*n* = 5], Asia [*n* = 3], Australia [*n* = 2] and Africa [*n* = 1]. Nationally, both USA and Finland produced three papers, while Sweden, United Kingdom [UK], Canada, China, and Australia had two papers each, and Pakistan and Rwanda had one paper each. The respondent sample size of all included papers ranged from 6 to 628. The papers that met the inclusion criteria spanned 2012 to 2022. [See Table 1].

### Thematic analysis

The analysis of the included studies generated five key themes: [i] knowledge and awareness of climate change, [ii] varying views on the link between nursing and climate change, [iii] environmental sustainability, [iv] barriers to environmentally responsible healthcare, and [v] routes to environmentally sustainable nursing practices.

### Knowledge and awareness of climate change

Knowledge and awareness levels varied amongst nurses as some studies reported moderate or low awareness level^12^. Regarding climate change, approximately half of Pakistani nurses were aware of this phenomenon [26]. Some staff nurses did not have any prior knowledge of the concept [17,27,28]. In the literature, some nurses were asked to define climate change and they equated it to global warming [28]. Some defined sustainability in healthcare as, ‘picking trash off the ground [29], or waste management [30]. Social media was the major route to information on climate change among nurses [17,26] while others reported TV news as their source [31]. The majority of nurses’ responses indicated a belief that humans are responsible for damaging the planet and causing climate change [15,26]. Others believed climate change is caused by both humans and natural changes [31]. Nurses recognised the impact of climate change on human health. Mental health disorders [15,29], climate refugees, immunocompromised people and infections [32], air pollution or air quality-related illness, asthmatic attacks [15,26,32], the effect on public health [17] and flooding [15,26] were all reported known impacts in nurses’ responses.

### Varying views on nursing and climate change link

It was reported in two studies that some nurses did not see the link between nursing practices, and climate change [17,29]. Where the connection was made between nursing activities and climate change, it was in relation to treating and caring for people affected by climate change, rather than acknowledging that healthcare and nursing practices contributed to carbon emissions and therefore had climate impact [29].

### Environmental sustainability

Sustainable practices in relation to climate change were reported by some nurses. for example; reducing consumption of goods [31], riding of bicycle to work [16]. In addition, statements on how to be sustainable were reported which included; effective use of material and energy, thus not throwing away unused bags from operation kits, and keeping a clean diaper when going on rounds [33]. Furthermore, technological advancement such as digitalisation reduces paper usage and using telehealth or remote consultations saves car trips hence reduces carbon emission [34]. These environmentally sustainable practices reportedly led to financial gains in the healthcare sector [30] and improvement in individual physical health [16].

### Barriers to environmentally responsible healthcare

The emphasis in nurses’ daily practice focused on meeting patients’ immediate needs, rather than specifically adopting environmentally responsible approaches to their work [16,35]. For example, nurses would not take actions during patient care activities which they might take at home for themselves – such as turning off a hot tap to save energy and risking their patient getting cold [33].

The literature suggests that policies supporting sustainability practices were poor or inadequate. In one study, waste bins were placed at the wrong locations, were the wrong sizes and a lack of colour coding limited the ability to segregate waste [33]. The dilemma between infection prevention actions and environmental sustainability is reported to impede sustainable healthcare practices [30]. Limited use of video-conferencing for staff meetings, and inadequate auditing of environmental sustainability were all reported [11] though this may have altered in more recent years since the advent of COVID-19 and the move to greater use of digital platforms and hybrid working.

Unessential procurement was reported to be one of the barriers to sustainable practices [33,35]. Some hospitals were not considering environmental sustainability in procurement decisions [11]. In the occurrence of weather disasters, there were reports of inadequate knowledge on how to face and support affected individuals [11,15]. In the light of adopting sustainable actions, some nurses were sceptical about their decision to be sustainable [27].

Organizational support for environmentally friendly practices was quite poor which meant that nurses could not willingly advocate for sustainable actions at the hospital [35]. Even with the desire to adopt practices to reduce waste and influence procurement, especially in the operating theater, nurses identified limited ability to achieve this because it did not appear to be a central concern of their employing healthcare organization [33,36].

### Route to environmentally sustainable nursing practices

There was the desire to be environmentally friendly among nurses [27,31]. Some were willing to learn more about climate change and sustainable nursing practices [17]. Many felt there was a responsibility to adopt green practice approaches [15], and these intentions could impact green behaviour [14]. Nurses suggested professional development as a means of increasing awareness of sustainability and green environmental practices [32,37]. Others agreed that staff training should include information on climate change [28,31,33].

## Discussion

This systematic review identified 18 papers from 9 countries across 5 continents. However, only one study was found in Africa, no studies in South America, and 3 in Asia. These are areas where the impact of climate change is higher or with a higher level of climate vulnerability or lack of readiness and resources to combat climate impact [38,39].

The major source of awareness of climate change among the nurses was the media. This finding is similar to the report in the Lancet Countdown 2022 [5,40]. This raises the question regarding the role of healthcare organizations, professional bodies, and educational institutions in relation to knowledge sharing, education, research and generating policy and professional practice guidance. As global pioneers in Net-Zero healthcare, the Centre for Sustainable Healthcare and Health Education England [HEE] launched courses on aspects of Greener NHS to educate NHS staffs on net-zero healthcare and a greener NHS [6]. Healthcare organizations can utilize this media to their advantage to propagate and provide the right information specifically tailored to nurses.

Humans are believed to be the major causes of climate change, and this is reaffirming the global consensus reached [41]. Recognition of the impact of climate on humanity appears to be an acknowledgment of the existence of climate change and can serve as a platform for a nursing response to the unfolding climate crisis [4]. The expression of ‘no link’ between nursing and climate change in some of the studies indicates the need to clarify nurses’ role in climate change, and how they can help reduce the impact.

The reviewed literature showed barriers to sustainability; for instance, nurses saw their primary duty to patients and this did not automatically align with green practices. This could be as a result of work overload or staff shortage. Inadequate implementation of policies, unessential procurement, inadequate preparedness of nurses, and lack of organizational support were some limitations noted. These barriers point to the need for healthcare organizations to include criteria related to environmental sustainability into procurement decisions and other aspects of service design and delivery. In the UK, connecting procurement decisions with sustainability is a relatively new concept but has recently been promoted by NHS England guidance [47] via the policy document, ‘Applying net zero and social value in the procurement of NHS goods and services’ [48]. Green approaches to procurement ensure that the materials and supplies ordered have the lowest carbon footprint possible. The involvement of nurses in greening procurement decision making will place nurses in a better position to identify the most environmentally friendly products for use in practice, and in designing commissioning briefs for services or equipment provision.

This finding could be significant for leaders when planning projects on climate sustainability or greening hospitals [42]. In the midst of these reported barriers, the review found the desire and readiness to be sustainable among nurses. This desire could be a driving force to increase the focus on nursing practice, its relationship with climate change, and its potential to reduce the healthcare carbon footprint. Education, professional development and curricula inclusion are known to have a positive impact on awareness levels, behavior and attitude change [28,31].

It is worth noting that the UK grey literature promotes the value of green champions and sustainability leads within the NHS. These roles could be held by nurses who could then take on a green advocacy role [49,50]. Recent approaches such as Royal College of Nursing (RCN) ‘Glove Awareness campaign’ and Centre for Sustainable Healthcare are helping to develop awareness and providing tangible options for nurses to make changes to their practices. The Glove Awareness campaign is an RCN initiative launched in 2018, and its week-long campaign aimed to encourage appropriate glove use by raising awareness of sustainability factors and the importance of good skin health [51]. The Sustainability in Quality Improvement framework (SusQI) is an approach to improving healthcare in a holistic way, by assessing quality and value through the lens of a “triple bottom line” [52], which encompasses environmental, social and economic cost and impact.

### Limitations

The language restriction to ‘English’ during the database search could have led to the possibility of missing some relevant papers and publication bias. However, most of the current scientific papers in science are published in English or at least have an abstract written in English in addition to the original language [43] and no study was excluded during the title or abstract screening due to language. Secondly, some of the studies did not have nurses primarily as the only subjects but included healthcare professionals; however, reported data derived from nurses alone were extracted from those studies. Finally, to capture more relevant studies, the search term was manipulated in the SCOPUS database to retrieve all the studies noted during the scoping searches.

### Strengths

To our knowledge and from our database search, this is the first systematic review which used a mixed-method approach to explore the review topic. The use of a mixed method approach gave a wider and deeper understanding of the topic [44]. Qualitizing was chosen to reduce the risk of error as opposed to quantitizing [45]. A prior review protocol was developed to help reduce the risk of biases and ensure trustworthiness, transparency, and accountability [46]. Finally, most of the studies had maximum or fair methodological quality by using the MMAT [22] and JBI qualitative appraisal tool [23].

## Conclusion

The review indicates the need to raise awareness regarding climate change and sustainable practices among nurses. It also reinforces the importance of clarifying and conscientizing the nursing professions’ role in finding solutions to combat climate change. The review makes a case for the use of professional development, in-service training, nursing competencies, and inclusion into curricula to enhance nurses’ knowledge of climate change and their role in combating it within professional practice. Furthermore, engagement with policy makers and healthcare leaders to ensure criteria relating to environmental sustainability are included in procurement decisions, the planning of new healthcare facilities, and design of services, with the involvement of nurses in these decisions, is recommended. Future research to explore the link between nursing practices in different settings and the carbon footprint is needed, and more emphasis on tailored education about climate change, sustainable healthcare and nursing is required.

## Ethics

Ethical approval [P140687] was obtained from the Ethics Committee, Coventry University, UK.

## Data sharing

The search strategy is available in the supplementary file. Any additional data are available upon request from the corresponding author.

## Funding

This PhD is funded by the Research Centre for Healthcare and Communities, Coventry University, UK.

## CRediT authorship contribution statement

**Ebenezer Akore Yeboah:** Conceptualization, Formal analysis, Methodology, Writing – original draft, Writing – review & editing, Software, Data curation. **Amanda Rodrigues Amorim Adegboye:** Conceptualization, Formal analysis, Methodology, Supervision, Writing – original draft, Writing – review & editing, Funding acquisition. **Rosie Kneafsey:** Conceptualization, Formal analysis, Supervision, Writing – original draft, Writing – review & editing, Funding acquisition.

## Declaration of competing interest

The authors declare that they have no known competing financial interests or personal relationships that could have appeared to influence the work reported in this paper.

## Appendix Supplementary materials

Image, application 1

Image, application 2
