# Supplementary material for: Nurses’ perceptions, attitudes, and perspectives in relation to climate change and sustainable healthcare practices: A systematic review
Source: J Clim Chang Health. 2023 Dec 2;16:100290. doi: 10.1016/j.joclim.2023.100290 (PMC12851204; doi:10.1016/j.joclim.2023.100290)
Supplement: Supplementary file 1 [file mmc1.docx]

**Supplementary material**

Table of content

Search term for one database …………………………………………………………………..2

Inclusion and exclusion criteria ………….……………………………………………………..2

Qualitative and mixed method findings……………………………………………………..4

Quantitative study findings …………….……………………………………………………..6

**Full search strategies for nurses’ perception, attitude, and perspective towards sustainability practices in relation to climate change.**

**Database: PubMed**

Search date: 1^st^ September, 2022 at 12:57

#1 "Nurses"[Mesh] OR "Nursing"[Mesh] OR "nurs*"[tw]

#2 "Climate Change"[Mesh] OR "Greenhouse Effect"[Mesh] OR "Carbon Footprint"[Mesh] OR "climate change"[tw] OR "sustainab*"[tw] OR "sustainable healthcare"[tw] OR "environmentally responsible healthcare"[tw] OR "global warming"[tw] OR "environmental responsib*"[tw] OR net zero healthcare [tw]

#3 "Survey*"[tw] OR " questionnaire*"[tw] OR "interview*"[tw] OR "observ*"[tw] OR "case stud*"[tw] OR "focus group*"[tw]

#4 "Social Perception"[Mesh] "Attitude"[Mesh] "Awareness"[Mesh] OR "Opinion*" OR "perce*"[tw] OR "aware*"[tw] OR "perspect*"[tw] OR "attitude*"[tw] OR "belie*"[tw] OR "know*"[tw] OR "pract*"[tw]

#5 ("Qualitative Research"[Mesh] OR "mixed method*"[tw] OR qualitative[tw] OR quantitative[tw]

#1 AND #2 AND #3 AND #4 AND #5

Filters: English

Results found: 702

**Table 2. Inclusion and exclusion criteria in the literature search**

| **Domains** | **Inclusion** | **Exclusion** |
| --- | --- | --- |
| Sample | - Studies/ publication /articles focused on registered or qualified professional nurses regardless of work setting - Can include other health professionals if findings reported nurses separately. - Studies across the globe or any country | - Studies/ publication /articles on climate change focusing on nursing students and/or nursing educators. - Studies on other healthcare professional e.g., care assistants. |
| Phenomenon of interest | - Climate change, environmental sustainability, net zero healthcare and nursing practice or any related terms to these | - Studies that do not include climate change or sustainable environmental practice or any related term |
| Design | - Empirical research studies using any research design e.g., interview, survey, focus group etc. - Published literature, governmental reports, technical documents, manuals, conference abstracts, non-governmental reports on net zero healthcare (or climate change) with relation to nursing (which are primary or empirical study). | - Commentaries, press releases, speeches, and letters to the editor are excluded - All publication on the phenomena of interest which are not empirical research. |
| Evaluation | - Studies exploring the perception, OR attitude, OR awareness (or knowledge) OR the perspective on climate change and related terms |  |
| Research type | - Qualitative, quantitative or mixed methods approach of primary research | - Secondary research |

**Table 3: Qualitative and mixed method study findings**

| **Author/**  **Year** | **Key findings** | **Quotes** |
| --- | --- | --- |
| Anaker, et al, (2015). | 2 themes:  (i) incongruence between climate issues’ and nurses’ daily work  (ii) Public health work as a health co-benefit of climate change mitigation. | *‘In a typical day, we do not have time to think about the environment’*  ‘Riding a bicycling to work is good for health and the environment.’ |
| Baid, et al, (2019). | Sustainability concerns carbon footprint and ecological damage.  Perception of the misuse of infection prevention actions and inability recycle physical waste products.  The interconnectivity of systems and the financial, environmental, and social aspects of care. | *‘Sustainability is … to reduce our footprint environmentally, whether it's from emissions or waste management, and looking at how we can take greater care of the resources ...’*  *‘Maintaining sustainability will improve the environment and will also be part of the efforts for saving money*’  *‘During a busy shift or when working in an isolation room, we stock up on materials not necessarily required for the patient*…’ |
| Kalogirou, et al (2020) | Main themes:  i. muddled terminology  ii. Climate change and health  iii. Nursing's relationship to climate change.  Varying levels of knowledge about climate change and its relationship to health or practice.  Climate change was a personal concern, but nursing's role in addressing it was not understood | *‘ It means obviously you would do things, you know, the amount of electricity you use, not littering,… helping out the environment in small ways, picking up a piece of trash off the ground’*  *‘To me, climate change is associated with the global warming due to the effects of man's influence on the environment’*  *‘… If you're used to a certain type of weather and you move somewhere for this type of reason and let's say it's raining every day, I think it affects people's mood… People's mental health can [be impacted]. It might affect their careers.’*  *‘I honestly don't see a link… I don't know how my nursing profession can affect [climate change] when I'm in a building that's climate-controlled and I'm asked to use the supplies that I have’*  *‘I have not encountered one single nurse who doesn't understand that it exists…’*  ‘*There is a connection where we try to combat the changes of climate change on our patients by the type of care that we provide for them, … That's our role…’* |
| Kalogirou, et al, (2021) | 3 themes:  i. Patient care not environmental care  ii. Organizational role  iii. Operational efficiency  Patient care was their primary priority, and due to their workload, they were unable to simultaneously consider the environmental impact of their work. | *‘I focus on patient need right then at that moment. If my patient needs four warm blankets to stay warm… I'm not thinking of those things down the road…’*  *‘We're here to serve the patient…’*  *‘…You have to go through so many hoops until you get to [the right] person [and] sometimes they say "no", even though you know it's going to positively change the work area’*  *‘A lot of the supplies that we get do not do the job properly, but because they're lower cost alternatives, that is what we get. What happens is people get frustrated, they throw it out, and they go find something else…’* |
| Iira, et al. (2021). | 3 main themes:  i. Identifying vulnerable groups to climate change  ii. Recognizing the impact of shifting seasons  iii. Lacking essential knowledge to provide evidence‐based care.  The nurses attributed some changes in their patients' health to climate change.  Nurses felt that basic nursing education and staff development do not focus enough on climate change. | *‘people are older, and they are more…they get easily infections if they are… And their immunology is low.’*  *‘I mean that is an effect of the climate change, you get climate refugees’*  *‘I have noticed that in springtime there is much more longer time dusty and many people are suffering…asthma people, allergic people…they can't breathe, they can't go out, they can no train…do training, because it's too dusty. This time gets longer and longer every spring’*  *‘I'd like to take course where you are talking about climate change, sort of where we have come to and what can be expected, it would be kind of linked to our preventive health care, those aspects you should consider.’* |
| Leppänen, et al, (2022). | The principles of sustainable development were poorly known among the participants.  Nurse managers considered their opportunities to influence decision- making were reduced by their limited economic knowledge.  Resource use, individuality, and ecological viewpoints were emphasised in the decision- making process in perioperative work. | *‘I´m not actively for recycling…maybe, if there would be better rubbish bins…But, I don´t know if that would make any difference either…’*  *‘Although, perioperative nurses were generally concerned about climate change, they did not see it as something they could influence at work’* |
| Koltsida, et al (2021). | The registered nurses experienced reduced consumption and damage to the environment;  The use of IT affected social aspects such as the work environment and patient safety, and positive consequences. | *“...calling a nurse and a doctor instead of taking the car saves a lot of trips...”*  *“... the more technical it becomes, the less paper I can say is used ...”* |
| Dunphy 2014 | A lack of professional development about environmental sustainability and healthcare contributes to these problems.  Environmental sustainability information was described in the context of the home, but not the workplace, and reported that professional development is required. | *‘I mean we’re all being educated at home about climate change and those sorts of things being again good stewards of the planet…or you have collections of people who have an interest, …, doing that together as part of their professional development’* |
| Kallio, et al, (2020) | Environmentally responsible clinical practices focus on effective material and energy use.  Material use focused on sustainable and cooperative purchasing, considerate use of products and minimising waste and idle electricity and reducing transport use.    The tools needed to promote environmental responsibility in clinical practice were staff inducements and training and certain resources and guidance.  All the hospital health and support service staff needed to commit to collaboration.  78% agreed that staff’s training should cover information on climate change.  ‘96% agreed that nurse managers ensure that staff have sufficient environmental competencies’  ‘89% agreed that hygiene nurses consider the environment when preventing infection’  ‘85% reported the significance to financially support the use of public transport’ | *“Nurses in my unit have said that it doesn't make sense to order certain products that we don't use anymore.”*  *“The waste bins are the wrong size and in the wrong places.” “All the waste bins look the same.”*  *‘I can use an unused bag from an operation kit to take samples to the laboratory instead of throwing it away’*  ‘*There is no space in the units for the waste bins’*  *‘A great shelf had been ordered full of the kind of intravenous tubes that we only use one a month…’*  *‘There is no need to change a diaper every shift if it is clean’*  *‘You can't turn off a tap and let the patient get cold while you wash their back in a shower’* |

***Data on nurses only were extracted from Baid et al, 2019 and Dunphy, 2014.***

***Data on ecological sustainability was extracted from Koltsida, et al, 2021 and Leppänen, et al, (2022).***

**Table 4: Quantitative study findings**

| **Author/year** | **Key findings** |
| --- | --- |
| Xiao et al. (2016) | 76% indicated climate change would affect public health.  24% lack relevant knowledge.  More than half of the nurses did not know their work could also affect the development of climate change.  83-96% were willing to learn the knowledge about climate change and sustainable nursing.  A majority of 32% had heard about climate change from social media and 45%, also a majority wanted to learn through the media. |
| Polivka, et al, (2012) | 75% agreed that humans are severely abusing the environment.  46% believed climate change was completely caused by humans.    Nurses (51.3%) were perceived as having responsibility to address health-related impacts of climate change but were unprepared (75.5%) or lacking the ability to address impacts (61.9%)  More than 50% reported vector-borne diseases, flooding-related displacement, mental health conditions, and air quality–related illnesses have increased due climate change |
| Buriro, et al (2018). | 50.5% have heard of climate change.  68.5% indicated humans are severely abusing the planet.  94.4% considered that air pollution affects their health.  70.5% reported that flooding as one of the adverse effects of climate change.  78% stated social media is their source of information.  Academic qualification showed an association with climate change perception (p=0.05). |
| Li, et al (2021). | Green behaviour intentions impacted employee green behaviour (r = 0:47, p < 0:01)  Individuals who set the goal to protect the environment and sustainable development should be more likely to show green behavior because setting a goal increases one’s inner motivation  Ethical leadership was positively related to employee green behaviour (r = 0:68, p < 0:01)  The intention to engage in green behaviour inﬂuences nurses’ green behaviour positively, and the relationship is stronger when ethical leadership is high in the organization (CE== 0:53, SE = 0:16, t = 3:38, p < 0:01, 95% confidence interval of 0.22- 0.84) |
| Schenk, et al, (2021) | Practicing nurses (M=3.54) were more concerned, had a better awareness score, higher motivation scale score on climate change as compared to students (M=3.30).  However, faculty nurses (M=3.77) were averagely better in the parameters than practicing nurses. |
| Barraclough, et al, (2019). | 18% and 10% use low energy lighting and renewable energy respectively.  20% had never performed an environmental sustainability audit  Only 27% of the hospitals centres reported regular use tele- or video-conferencing to replace staff meetings.  39% considered environmental sustainability in procurement decisions  There was minimal preparedness to cope with climate change. |
| May et al, 2019 | 65% said climate change is caused by both human activities and natural changes.  “I believe I can contribute to the solution of environmental issues by my actions” (n = 33/40)  “I make an effort to reduce the amount of goods I consume” (n = 33/40)  Top source of knowledge was TV news (29%) and internet source (21%)  Knowledge has a positive impact on attitudes and behaviours in school nurses. |
| Nieto-Cerezo, 2016 | Nurse staff contribution represents up to 47 % of staff total emissions  53% of nursing professionals were positive or keen about the interest free public transport season ticket loan scheme initiative to reduce carbon emission.  17% of nursing professionals chose the car sharing scheme but had some degree of scepticism around reliability and potential relational issues.  Some staff did not have any prior knowledge of the sustainable healthcare transport policies |
| Nsengiyumva et al, 2020 | 35.6% and 20.9% had low and mild awareness on climate change and neonatal health risks.  Education level and high awareness level on climate change and associated neonatal health risks were significantly related(p=<0.05) |

***Data on nurses were extracted from Schenk et al, 2021 and Nieto-Cerezo, 2016.***
