# Supplementary material for: Nurses’ perceptions, attitudes, and perspectives in relation to climate change and sustainable healthcare practices: A systematic review
Source: J Clim Chang Health. 2023 Dec 2;16:100290. doi: 10.1016/j.joclim.2023.100290 (PMC12851204; doi:10.1016/j.joclim.2023.100290)
Supplement: Supplementary file 2 [file mmc2.pdf]

Certificate of Ethical Approval

Applicant:	Ebenezer Akore Yeboah
Project Title:	PERCEPTION, ATTITUDE, AWARENESS AND PERSPECTIVE TOWARDS SUSTAINABILITY PRACTICES IN RELATION TO CLIMATE CHANGE AMONG NURSES. A SYSTEMATIC REVIEW.

This is to certify that the above named applicant has completed the Coventry University Ethical Approval process and their project has been confirmed and approved as Low Risk

Date of approval:	16 Dec 2022
Project Reference Number:	P140687
